# Dihydroorotate dehydrogenase inhibition acts synergistically with tyrosine kinase inhibitors to induce apoptosis of mantle cell lymphoma cells

**DOI:** 10.1002/jha2.434

**Published:** 2022-05-15

**Authors:** May Eriksen‐Gjerstad, Ida Tveit Karlsen, Zinayida Fandalyuk, Susanne Benjaminsen, Fanny Baran‐Marszak, Bela Papp, Frederick Locke, Marcus Ladds, Andrés Pastor‐Fernández, Pascal Gelebart, Emmet Mc Cormack

**Affiliations:** ^1^ Department of Clinical Science University of Bergen Bergen Norway; ^2^ Inserm U978 Université Paris‐13 Bobigny France; ^3^ Institut National de la Santé et de la Recherche Médicale UMR U976 Institut de Recherche Saint‐Louis Hôpital Saint‐Louis, Université de Paris; CEA DRF‐Institut Francois Jacob Department of Hemato‐Immunology Research Hôpital Saint‐Louis Paris France; ^4^ Department of Blood and Marrow Transplant and Cellular Therapy Moffit Cancer Centre Tampa USA; ^5^ Department of Microbiology Tumor and Cell Biology (MTC) Karolinska Institutet Stockholm Sweden; ^6^ SciLifeLab Department of Microbiology Tumor and Cell Biology (MTC) Karolinska Institutet Stockholm Sweden; ^7^ Department of Clinical Science University of Bergen Bergen Norway; ^8^ Department of Quality and Development Hospital Pharmacies Enterprise in Western Norway Bergen Norway; ^9^ Centre for Cancer Biomarkers CCBIO Bergen Norway

**Keywords:** apoptosis, DHODH, mantle cell lymphoma, targeted therapy

## Abstract

Mantle cell lymphoma (MCL) is a non‐Hodgkin lymphoma that remains incurable with the treatment options available today. In the present study, we have identified the dihydroorotate dehydrogenase (DHODH), an essential enzyme for the *de novo *biosynthesis of pyrimidine‐based nucleotides, to be overexpressed in MCL in comparison to healthy peripheral blood mononuclear cells (PBMC). In vitro inhibition of the DHODH activity using a newly developed DHODH inhibitor, namely (*R*)‐HZ05, can induce MCL cell death in the nanomolar range independently than the P53 status of the investigated cell lines. Moreover, the combination of (*R*)‐HZ05 with tyrosine kinase inhibitor shows the synergistic activity on cell death. Pre‐clinical investigation on the efficacy of (*R*)‐HZ05 shows that it can be prolonged animal lifespan similar to ibrutinib. (*R*)‐HZ05 use in combination with tyrosine kinase inhibitor demonstrated a superior efficacy on tumor burden reduction and survival than either drug alone. We have demonstrated that the depletion of the pyrimidine nucleotide pool, using DHODH inhibitor, represents a new therapeutic strategy that may benefit MCL patients.

## 1 INTRODUCTION

Mantle cell lymphoma (MCL) is a non‐Hodgkin lymphoma which remains incurable with the treatment options available today. Despite the good response rate to upfront chemotherapy, most patients relapse, presenting with an overall survival of 3–5 years [1, 2, 3, 4]. The majority of MCL patients have the *t*(11;14)(*q*13;*q*32) translocation which leads to a deregulation of the cell cycle through the overexpression of cyclin D1 [5]. Along with oncogenic alterations in different cellular pathways (STAT3, NF‐κB, AKT, Wnt, INK4a‐CDK4‐RB1) [2, 3], the deregulation of the cell cycle leads to an aggressive and highly proliferative disease that demands a constant supply of nucleotides for RNA transcription and DNA replication [1]. Targeting metabolic pathways is one of the most successful strategies in anti‐cancer therapies [6]. Recently, Ladds et al. characterized a novel dihydroorotate dehydrogenase (DHODH) inhibitor, namely (*R*)‐HZ05, that increases the sensitivity to chemotherapy in acute myeloid leukemia (AML) cells [7]. DHODH is an essential enzyme for the *de novo* biosynthesis of pyrimidine‐based nucleotides [6]. DHODH catalyses the conversion of dihydroorotate into orotate, which in turn is converted through several catalytic steps into deoxycytidine triphosphate, deoxythymidine triphosphate, and deoxyuridine triphosphate. Inhibition of DHODH has been linked to S‐phase arrest and apoptosis induction through p53 upregulation [7] and his regarded as a new therapeutic target in cancers [8, 9]. Importantly, as in AML, many MCL patients present with wild‐type p53 [10, 11]. Therefore, we hypothesize that targeting DHODH activity in MCL, using (*R*)‐HZ05, as a single‐agent and as a combinatory agent, represents a promising and yet unexplored strategy in MCL.

## 2 RESULTS AND DISCUSSION

**FIGURE 1:**
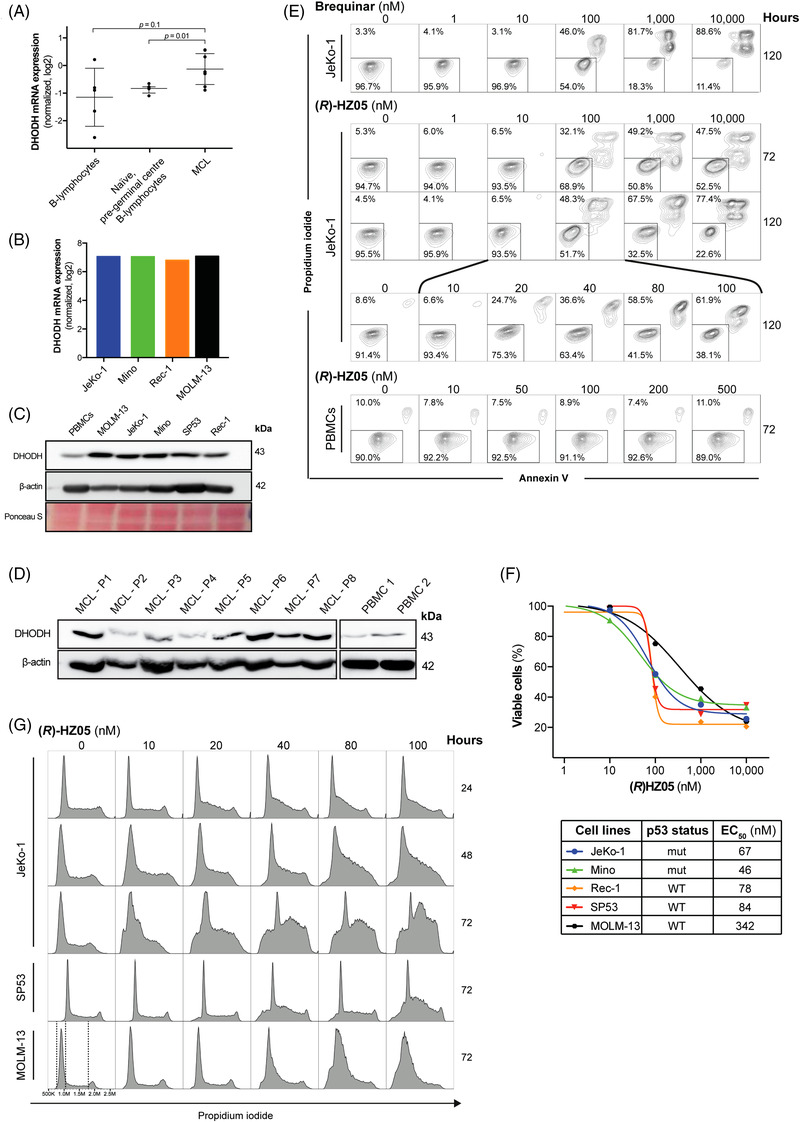
(A) Normalized dihydroorotate dehydrogenase (DHODH) mRNA expression levels in B‐lymphocytes, naïve pre‐germinal center B‐lymphocytes and primary mantle cell lymphoma (MCL) cells. (B) Normalized DHODH mRNA expression levels in MCL and acute myeloid leukemia (AML) (MOLM‐13) cell lines. (C) Protein expression level in PBMCs, MOLM‐13, and the MCL cell lines JeKo‐1, Mino, SP53, Rec‐1, and in (D) various primary MCL cells. β‐actin and Ponceau staining were used as loading controls. (E) JeKo‐1 cell viability analyzed by AnnexinV/PI flow cytometry following treatment with 1–10,000 nM brequinar (120 h) or (*R*)‐HZ05 (72 h and 120 h), and following treatment with 10–100 nM (*R*)‐HZ05 (120 h). Cell viability of healthy PBMCs using 0–500 nM (*R*)‐HZ05 (72 h). (F) Dose–response curves and estimated EC_50_ values of MCL cell lines and MOLM‐13, treated with 1–10,000 nM of (*R*)‐HZ05 for 120 h. All results determined by AnnexinV/PI Flow Cytometry. (G) Cell‐cycle analysis of JeKo‐1, SP53 and MOLM‐13 treated with 10–100 nM (*R*)‐HZ05 (72 h) determined by flow cytometry using propidium iodide. JeKo‐1 was also analyzed after 24 and 48 h with (*R*)‐HZ05 exposure

Data collected from the Oncomine database indicate that normalized DHODH mRNA expression levels in MCL patient cells (*n* = 8) are upregulated compared to B‐lymphocytes collected from healthy donors (*n* = 5, *p* = 0.1) and significantly upregulated compared to naïve, pre‐germinal center B‐lymphocytes from healthy donors (*n* = 5, *p* = 0.01; Figure 1A). Supporting this finding, data from the Cancer Cell Line Encyclopaedia show that the normalized DHODH mRNA expression level of various MCL cell lines (JeKo‐1, Mino and Rec‐1) are similar to the DHODH mRNA level observed in the AML cell line MOLM‐13 (Figure 1B). Also, the protein expression of DHODH is higher in MCL cell lines (Figure 1C) and MCL primary cells (Figure 1D) when compared to PBMCs from healthy donors. Collectively, these observations suggest that DHODH is a potential target for inhibition in MCL.

**FIGURE 2:**
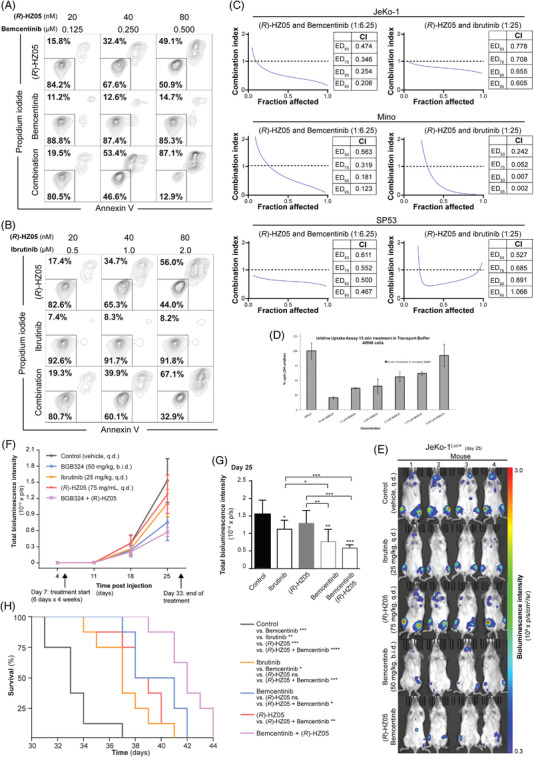
(A) AnnexinV/PI cell viability assay of JeKo‐1 cells following (*R*)‐HZ05 treatment (20, 40, or 80 nM) for 120 h, bemcentinib treatment (0.125, 0.250, or 0.500 μM) for 48 h, or (*R*)‐HZ05 and bemcentinib in combination (ratio 1:6.25). Bemcentinib was added to the cells 72 h after the adding of (*R*)‐HZ05. (B) AnnexinV/PI cell viability assay of JeKo‐1 cells following (*R*)‐HZ05 treatment (20, 40, or 80 nM) for 120 h, Ibrutinib (0.5, 1.0, and 2.0 μM) for 48 h, or (*R*)‐HZ05 and Ibrutinib in combination (ratio 1:25). Ibrutinib was added to the cells 72 h after adding of (*R*)‐HZ05. **(C)** The calculated synergistic effect of combining (*R*)‐HZ05 with bemcentinib or Ibrutinib in JeKo‐1, Mino, and SP53. The synergistic effect is illustrated by the combination index (CI) vs. fraction affected, and CI vs. effective dose (ED) required to affect 50% (ED_50_), 75% (ED_75_), 90% (ED_90_), and 95% (ED_95_) of the cells. (D) Uridine Uptake Assay in the melanoma cell line ARN8. (E) Total bioluminescence intensity at day 25 of a JeKo‐1*^Luc+^* xenograft model after treatment with single‐agent or combinations of bemcentinib, (*R*)‐HZ05 and ibrutinib. The model was established on day 0, randomized at day 4, and treated from day 7, 6 days × 4 weeks with either vehicle (q.d.), 50 mg/kg (b.i.d.) bemcentinib, 75 mg/kg (q.d.) (*R*)‐HZ05, 25 mg/kg (q.d.) Ibrutinib, or combination of bemcentinib and (*R*)‐HZ05. The combination group received the same dose as the single treated group. Bioluminescence imaging (BLI) was assessed on days 4, 11, 18, and 25 from the dorsal and ventral side (*n* = 8), using 50 mg/kg *D*‐luciferin. Representative images at day 25 from respective mice of each group are used to make the figure (F). Total bioluminescence intensity over time (G). Total bioluminescence intensity signal at day 25. Statistical significance compared to control is marked with a star above the column (*p < 0.05, **p < 0.01, ***p < 0.001, ****p < 0.0001). All comparisons with significance are marked, all other comparisons are non‐significant (ns). Error bars show standard deviation. (H) Kaplan–Meier curve presenting the overall survival from the different treatments

To determine the possible therapeutic value of inhibiting DHODH in MCL, we investigated the effect of the novel DHODH inhibitor (*R*)‐HZ05 on cell viability in vitro in comparison to brequinar a previously reported DHODH inhibitor. Inhibition of DHODH using (*R*)‐HZ05 or brequinar induces apoptosis in MCL cell lines in the nanomolar range (Figure 1E). In contrast, (*R*)‐HZ05 treatment does not affect PBMC viability. Interestingly, the EC_50_ values for the different MCL cell lines demonstrate that the p53 mutational status does not correlate with the sensitivity to (*R*)‐HZ05 (Figure 1F). This indicates that DHODH inhibition may be independent of p53 function despite its expression being upregulated in p53 positive cell lines Rec‐1 and SP53 (Figure S1A). It demonstrated that (*R*)‐HZ05 induces S‐phase cell accumulation in AML cell lines. To investigate if (*R*)‐HZ05 induce S‐phase arrest in MCL, we examined the effect of (*R*)‐HZ05 on the cell cycle in the MCL cell lines JeKo‐1 and SP53. The S‐phase cell accumulation was detectable after 24 h of (*R*)‐HZ05 exposure at concentrations ranging from 20 to 100 nM (Figure 1G). After 48 and 72 h, the S‐phase cell accumulation was more prominent (Figure S2A–C). Cell‐cycle arrest was accompanied by apoptosis (Figure S1B), as a time‐ and concentration‐dependent cleavage of PARP was observed following DHODH inhibition by (*R*)‐HZ05 in MCL cells. Interestingly, DHODH inhibition led to the up‐regulation of the DHODH protein in 2 of the 4 cells line tested (Figure S1C–F). This observation seems to indicate that DHODH is important for the maintenance of the MCL phenotype. We also observed a downregulation of cyclin D1, p27^Kip1^ and β‐catenin protein expression levels in MCL cells (Figure S1C–F). Notably, NF‐κB activation was not altered following (*R*)‐HZ05 treatment (Figure S1C, D). We thus hypothesized that subsequent inhibition of DHODH and NF‐κB signaling, an important signaling pathway for the proliferation and growth of MCL cells, might be of therapeutic value [12]. Since it has been suggested that inhibition of DHODH increases the chemosensitivity of cancer cell lines, we decided to investigate the effect of combining DHODH inhibition with a tyrosine kinase (TK) inhibitor that block NF‐kB activation [6]. We tested ibrutinib, a Burton tyrosine kinase (BTK) inhibitor already used in relapsed MCL patients, as well as bemcentinib, an AXL inhibitor in clinical trials for various cancers including AML, in combination with (*R*)‐HZ05. JeKo‐1 cells were treated with 20, 40, or 80 nM (*R*)‐HZ05 for 120 h in combination with either 0.5, 1.0, or 2.0 μM ibrutinib or 0.125, 0.250, or 0.5 μM bemcentinib. Both ibrutinib and bemcentinib were added to the cells 72 h after the addition of (*R*)‐HZ05, and the cells were further incubated for 48 h before analysis. Flow cytometry data indicate that the combination of bemcentinib and (*R*)‐HZ05 induces apoptosis in a synergistic manner (Figure 2A). While 80 nM (*R*)‐HZ05 for 120 h results in 49.1% apoptotic cells and 0.5 μM bemcentinib for 48 h results in 14.7% apoptotic cells, the combination results in 87.1% apoptotic cells. In comparison, the effect of combining ibrutinib with (*R*)‐HZ05 is rather additive, as 80 nM (*R*)‐HZ05 gave 56.0% apoptotic cells, and 2.0 μM ibrutinib gave 8.2% apoptotic cells, the combination gave 67.1% apoptotic cells (Figure 2B). The combinatorial effect between (*R*)‐HZ05 and bemcentinib or ibrutinib was evaluated, for the JeKo‐1, Mino, and SP53 cell lines, using the combination index (CI) versus the fraction affected for the JeKo‐1, Mino, and SP53 cell lines (Figure 2C). The data collectively show that the MCL cell lines are sensitive to the combination of either (*R*)‐HZ05 and bemcentinib or (*R*)‐HZ05 and ibrutinib.

It has been reported that some TK inhibitors can reduce the intracellular uptake of uridine in a dose‐dependent manner [13]. To evaluate if bemcentinib can prevent uridine uptake in cells, which may explain the synergism seen in combination with (*R*)‐HZ05, we performed a uridine uptake experiment. The uridine uptake in the melanoma cell line ARN8 is greatly reduced in a concentration‐dependent manner when treated with bemcentinib, suggesting that TK inhibition may prevent uridine transport also in MCL cells (Figure 2D).

(*R*)‐HZ05 was evaluated in vivo as a single‐agent and in combination with bemcentinib, compared to ibrutinib as a single‐agent, using a JeKo‐1*^Luc+^* MCL xenograft model. Four days after intravenous inoculation of JeKo‐1*^Luc+^* cells, the mice were randomized by bioluminescence intensity using 150 mg/kg *D*‐luciferin (*n* = 8). At day 7, treatment was initiated and continued for 6 days × 4 weeks using 75 mg/kg (*R*)‐HZ05 (QD), 50 mg/kg bemcentinib (b.i.d.), 25 mg/kg ibrutinib (q.d.) or the combination of (*R*)‐HZ05 and bemcentinib. Bioluminescence imaging was used to monitor the therapeutic efficiency, using 150 mg/kg *D*‐luciferin. The total bioluminescence intensities at day 25 indicate that (*R*)‐HZ05 as a single‐agent does not reduce tumor burden compared to vehicle (Figure 2E–G). In combination with bemcentinib, tumor burden is significantly reduced compared to the control group. The survival of the mice treated with (*R*)‐HZ05 alone or in combination with bemcentinib is significantly longer compared to the control (Figure 2H). Additionally, the overall survival of the group receiving the combination therapy is significantly longer than that of the group receiving (*R*)‐HZ05 as a single‐agent.

To evaluate the specificity of (*R*)‐HZ05 against DHODH, we explored the effect of uridine addition on DHODH inhibition in MCL cells. The addition of dihydroorotate (DA), a compound located upstream of the DHODH enzymatic activity, was not able to prevent the effect of DHODH inhibition in JeKo‐1 cells (Figure S3A, B). However, adding uridine rendered the cells resistant to (*R*)‐HZ05, suggesting that (*R*)‐HZ05 acts specifically on DHODH inhibition in MCL (Figure S3A–C). To evaluate the possible effect of the physiological uridine concentration present in the human body, we also performed a DHODH inhibition experiment in combination with TK inhibitor in the presence of 5 μM uridine. The addition of low concentrations of uridine in the experimental setting did not prevent the synergistic apoptosis‐inducing effect observed in MCL cells by the concomitant use of a DHODH inhibitor and a TK inhibitor (Figure S3D).

In conclusion, we demonstrate that DHODH is overexpressed at the mRNA and protein level in MCL primary cells and cell lines, compared to PBMCs. Our results show that the DHODH specific inhibitor (*R*)‐HZ05 can induce apoptosis of MCL cells as a single‐agent and act synergistically in combination with the AXL inhibitor bemcentinib. Interestingly, the p53 mutational status of MCL cells had no impact on the sensitivity to (*R*)‐HZ05 suggesting that the apoptosis induced by (*R*)‐HZ05in MCL cells is p53 independent. Also, DHODH inhibition induces a strong S‐phase cell accumulation of MCL cells, followed by apoptosis. This observation indicates that the apoptotic effect of (*R*)‐HZ05 is caused as a result of pyrimidine starvation. Our in vivo study shows that the (*R*)‐HZ05 is well tolerated in our MCL xenograft model. DHODH inhibition can increase the animal survival to the same extent than ibrutinib treatment. Interestingly, this can be achieved despite the observation that DHODH inhibition can led to an up‐regulation of DHODH (Figure S1D,E) and may have represent a resistance mechanism to the treatment. The co‐administration of (*R*)‐HZ05 with bemcentinib significantly prolongs survival in a mouse MCL xenograft model when compared to mice treated with vehicle, bemcentinib, ibrutinib, or (*R*)‐HZ05. Clinical trials with the newly developed DHODH inhibitor (*R*)‐HZ05 alone, or in combination with bemcentinib, are warranted in relapsed/refractory MCL patients.

## CONFLICT OF INTEREST

The authors declare that there is no conflict of interest that could be perceived as prejudicing the impartiality of the research reported.

## AUTHOR CONTRIBUTIONS

May Eriksen‐Gjerstad, Ida Tveit Karlsen, Pascal Gelebart, and Emmet Mc Cormack designed the experiments, May Eriksen‐Gjerstad, Ida Tveit Karlsen, Susanne Benjaminsen, Marcus Ladds, Andrés Pastor‐Fernández, Zinayida Fandalyuk, and M.P. performed all the experimental procedures, Pascal Gelebart, May Eriksen‐Gjerstad, Ida Tveit Karlsen and Emmet Mc Cormack analyzed the data. Frederick Locke, Bela Papp, and Fanny Baran‐Marszak provided MCL patients. All authors contributed to the writing of the paper.

## ETHICAL APPROVAL AND CONSENT TO PARTICIPATE

The use of human tissues and animal experiments have been approved by the Norwegian (REK number 2012/2245) and French institutional ethics committees.

## Supporting information

SUPPORTING INFORMATION

## Data Availability

The data that support the findings of this study are available from the corresponding author upon reasonable request.
