# Supplementary material for: Dihydroorotate dehydrogenase inhibition acts synergistically with tyrosine kinase inhibitors to induce apoptosis of mantle cell lymphoma cells
Source: EJHaem. 2022 May 15;3(3):913–8. doi: 10.1002/jha2.434 (PMC9422018; doi:10.1002/jha2.434)
Supplement: Supplementary file 1 — SUPPORTING INFORMATION [file JHA2-3-913-s001.docx]

SUPPLEMENTARY INFORMATION

“**The newly developed dihydroorotate dehydrogenase inhibitor (*R*)-HZ05, acts synergistically with tyrosine kinase inhibitors to induce apoptosis of mantle cell lymphoma cells”**

**TABLE OF CONTENTS**

SUPPLEMENTARY FIGURES AND LEGENDS 2

METHODS 4

**Supplementary figures and legends**


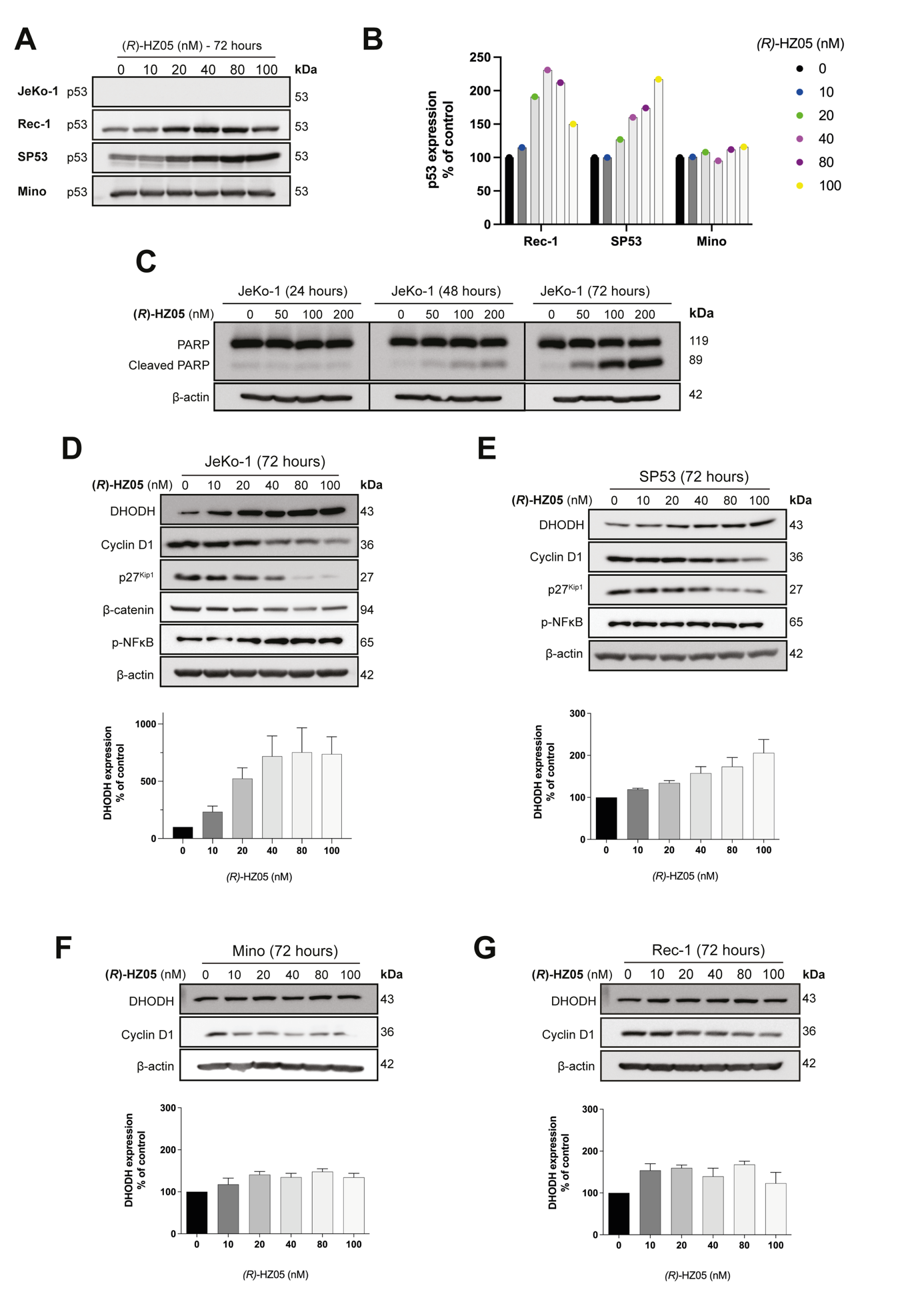


**Figure S1: Probing the molecular mechanism of DHODH inhibition in MCL cell lines.** MCL cell lines were treated with increasing concentrations of (*R*)-HZ05 for various treatment periods, and the cell lysates were evaluated by western blotting to determine the relative protein expression of target proteins of interest. β-actin protein expression levels were assessed to ensure comparable loading of protein samples. **(A)** Western blot analysis of relative protein expression levels of p53 in JeKo-1, Rec-1, SP53 and Mino, with increasing amount (0-100 nM) of (*R*)-HZ05 for 75 hrs. **(B)** Quantification of p53 protein expression level after (*R*)-HZ05 treatment in MCL cell lines. DHODH protein expression increases in REC-1 and SP53 cells. In contrast, p53 level doesn´t increase in Mino cells, a cell line that strongly expressed p53 at basal level (Amin*, et al* 2003) **(C)** Relative protein expression level of full-length PARP and cleaved PARP in JeKo-1 following treatment with (*R*)-HZ05 (50, 100 and 200 nM) for 24, 48 and 72 hrs. (**C**) Western blot evaluation of the protein levels of DHODH, cyclin D1, p27^Kip1^, β-catenin and p-NFκB in JeKo-1 cells following treatment of (*R*)-HZ05 (0-100 nM) for 72 hours. (**D**) Protein levels of DHODH, cyclin D1, p27^Kip1^ and p-NFκB in SP53 following (*R*)-HZ05 treatment (0-100 nM; 72 hours). Assessment of relative DHODH and cyclin D1 protein expression levels in Mino **(E)** and Rec-1 **(F)** following (*R*)-HZ05 (0-100 nM) treatment for 72 hours. The expression of DHODH increases after (*R*)-HZ05 treatment in 2 of 4 MCL cell lines tested.

**
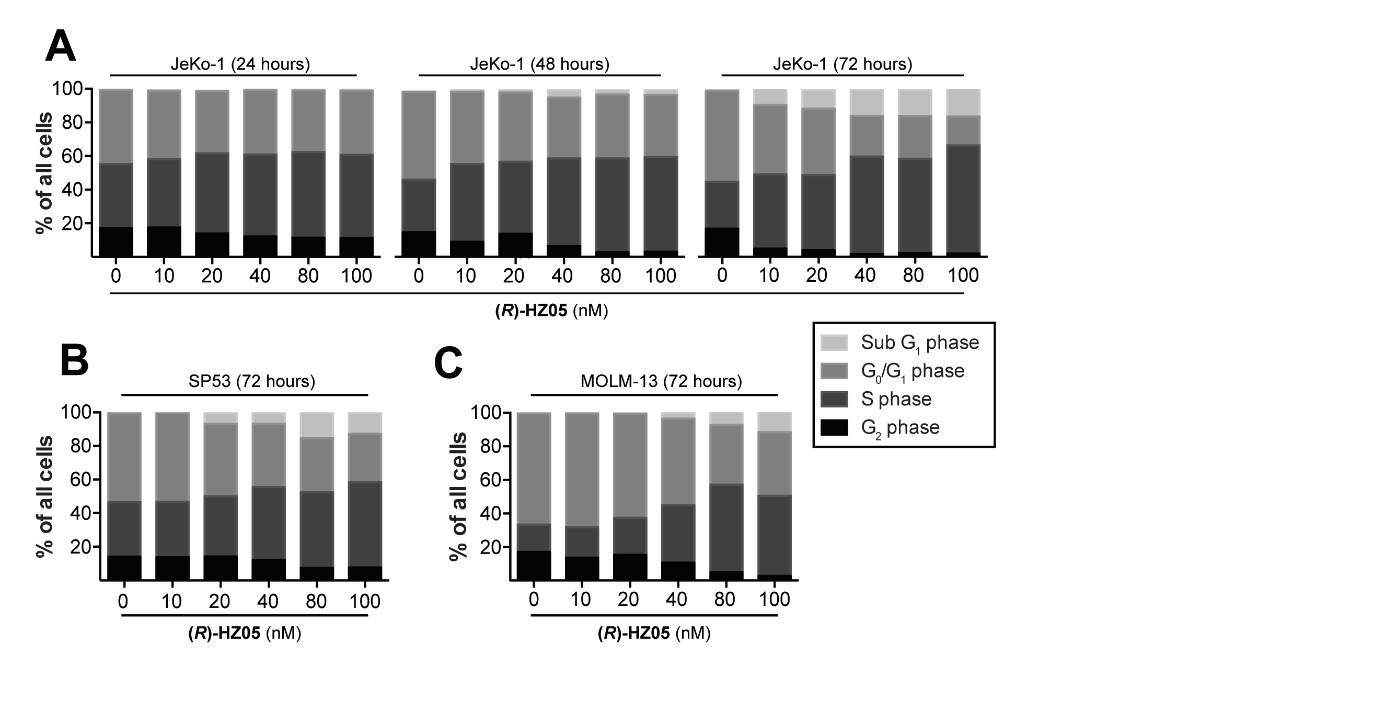
**

**Figure S2: Cell cycle arrest.** Cell cycle analysis of JeKo-1, SP53 and MOLM-13 treated with 10 – 100 nM (*R*)-HZ05 (72 hrs) determined by Flow Cytometry using propidium iodide. JeKo-1 was also analysed after 24 and 48 hrs with (*R*)-HZ05 exposure. The percentage of cells in each phase of the cell cycle based on the Flow Cytometry data, for **(A)** JeKo-1, **(B)** SP53 and **(C)** MOLM-13.


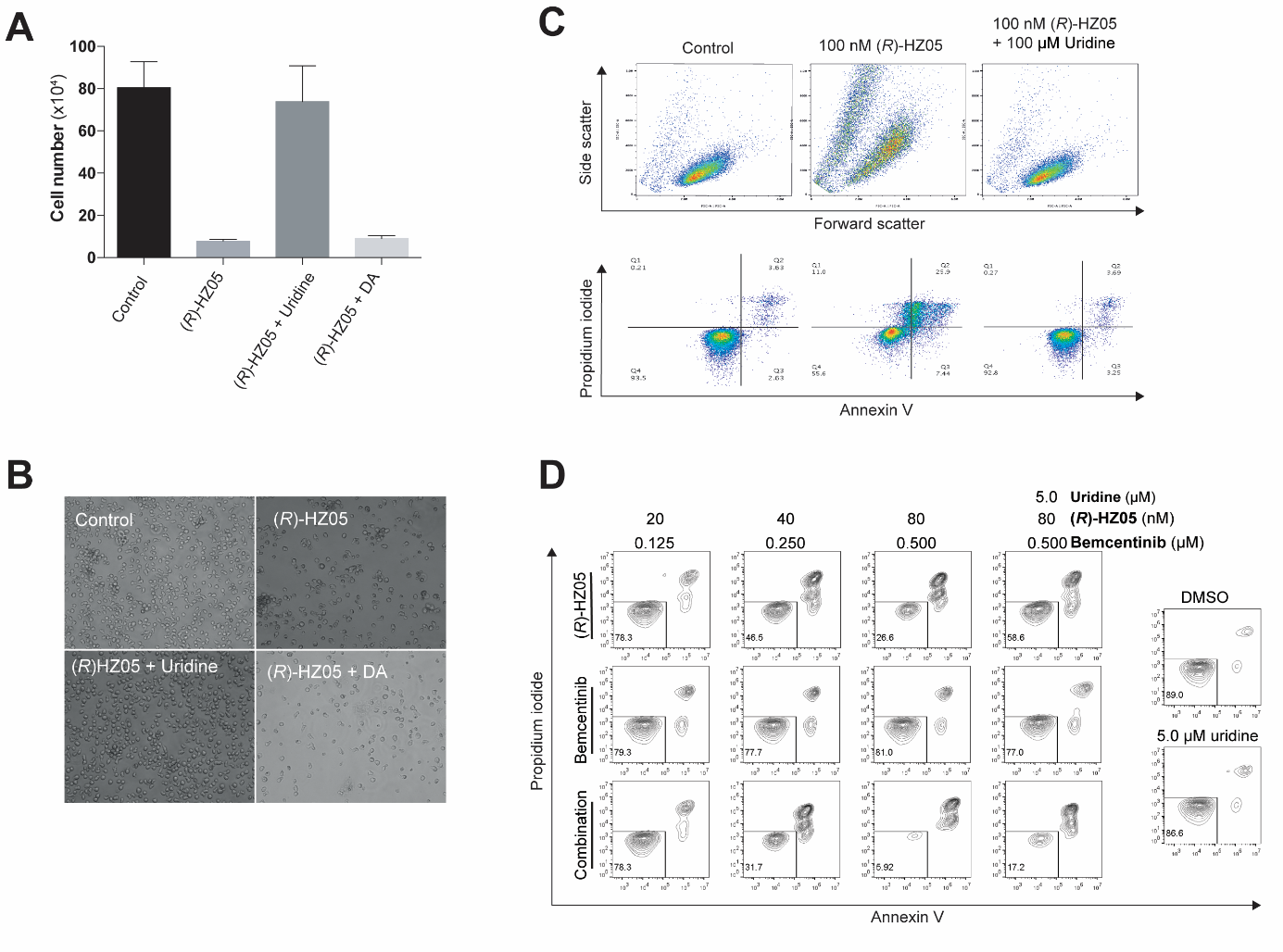


**Figure S3: Uridine experiments. (A, B, and C)** The effect of adding uridine on (*R*)-HZ05’s ability to block DHODH in JeKo-1 cells was evaluated by trypan blue cell counting after 72 hrs incubation with either DMSO (control), (*R*)-HZ05 alone, (*R*)-HZ05 and uridine or (*R*)-HZ05 and DA, an upstream substrate of DHODH. **(D)** JeKo-1 was treated with (*R*)-HZ05 (20, 40, 80 nM) or Bemcentinib (0.125, 0.250, 0.500 μM), or the combination of the two drugs. Additionally, at the highest concentration of the drugs as single agents or in combination, the effect of 5.0 μM uridine was evaluated. Control: DMSO or 5.0 μM uridine. Samples were analysed by AnnexinV/PI flow cytometry after 72 hrs incubation.

**
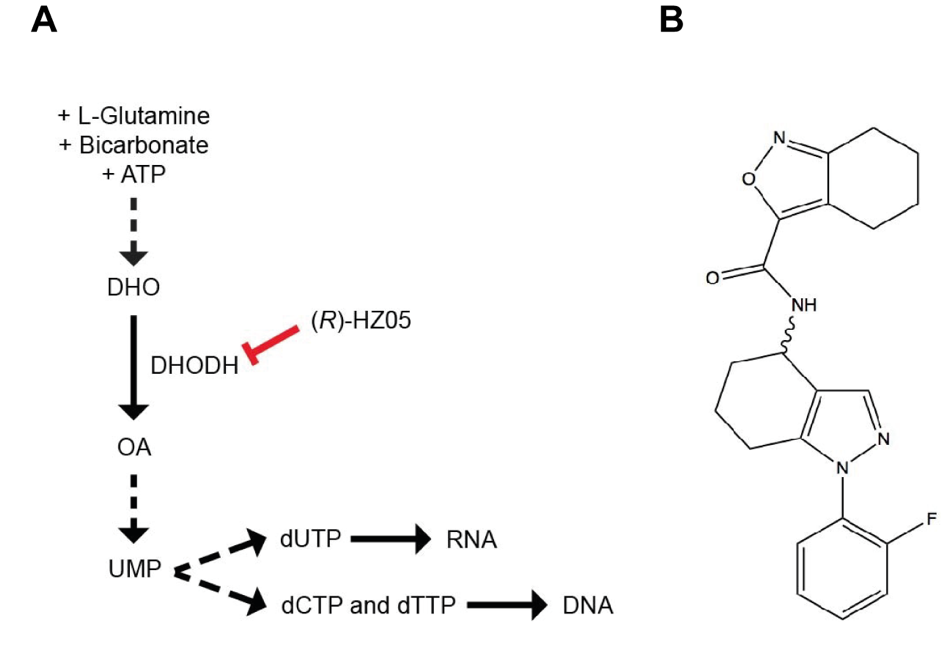
**

**Figure S4: DHODH pathway. (A)** (*R*)-HZ05 is an inhibitor of DHODH. **(B)** Chemical structure of (*R*)-HZ05.

**Methods**

**Cell lines, primary MCL cells and peripheral blood.** The human MCL cell lines JeKo-1, Mino, Rec-1 and SP53 were purchased from DZMZ (Deutsche Sammlung von Mikroorganismen und Zellkulturen GmbH, Braunschweig, Germany). The AML cell line MOLM-13 was bought from ATCC (American Type Culture Collection, Manassas, VA, USA). JeKo-1 cells expressing luciferase (JeKo-1*^Luc+^*) were generated using RediFect Red-FLuc-GFP lentiviral particles (PerkinElmer, Inc, Waltham, MA, USA). The expression of luciferase was confirmed by flow cytometry. All cell lines were cultured in RPMI-1640 medium (Invitrogen Corp, Carlsbad, CA, USA) supplemented with 10% (v/v) heat-inactivated foetal bovine serum (PPA Laboratories, GmbH, Pasching, Austria), 2 mM L-Glutamine and 50 U/mL penicillin-streptomycin (Sigma-Aldich, Inc, St Louis, MO, USA). Cells were seeded at at 1x10^5^ cells/mL (SP53; 2x10^5^ cells/mL) for *in vitro* experimentation. Mononuclear cells were isolated from peripheral blood using a density gradient separation (Lymphoprep, Nycomed Pharma AS, Oslo, Norway) in accordance with the manufacturer’s procedure. Mononuclear cells were seeded at 2x10^5^ cells/mL for *in vitro* experimentation. MCL primary cells were obtained from patients diagnosed at the Haukeland University Hospital (Bergen, Norway) and from the biobank of the Avicenne Hospital (Bobigny, France). The diagnosis was based on the World Health Organization Classification Scheme. All cases were confirmed to express cyclin D1 by immunohistochemistry. The use of these tissues has been approved by the Norwegian and French institutional ethics committees (REK number 2012/2245).

**Compounds.** (*R*)-HZ05 was synthesized and kindly donated by Professor Sonia Lain (Karolinska Institute, Stockholm, Sweden). Bemcentinib was ordered from CAERULUM (Shangai, China) for in vivo studies and Selleckchem (Houston, TX, USA) for in vitro studies. Ibrutinib was purchased from Selleckchem (Houston, TX, USA). Uridine, orotic acid monohydrate and DL-dihydroorotic acid were purchased from Sigma-Aldrich. All compounds were dissolved in DMSO and stored at -80°C for *in vitro* use. The final concentration of DMSO never exceeded 0.1% (v/v).

**Western blot.** Cells were lysed in RIPA Lysis and Extraction Buffer supplemented with 1% (v/v) Halt^TM^ Protease and Phosphatase inhibitor Cocktail 100X and 5 mM EDTA (Thermo Fisher Scientific, Waltham, MA, USA). The protein concentration was measured by DC Protein assay (Bio-Rad Laboratories, Hercules, CA, USA) following the manufacturer’s procedure. The following antibodies were used for western blotting: DHODH (generously donated by Dr. Bořivoj Vojtěšek, Masaryk Memorial Cancer Institute, Brno); p53 (Santa Cruz Biotechnology, Dallas, TX, USA; Cat. No. sc-263); β-actin (Cat. No. 4970), PARP (Cat. No. 9542), cyclin D1 (Cat. No. 2978), p27^Kip1^ (Cat. No. 2553), p-NFκB (ser536; Cat. No. 3033) and β-catenin (Cat. No. 9562) were all purchased from Cell Signalling Technology, Inc. (Danvers, MS, USA). All primary antibodies were diluted 1:1000. SuperSignalTM West Pico Chemiluminescent Substrate (Thermo Fisher Scientific, Waltham, MS, USA) was used to induce and enhance chemiluminescence. The chemiluminescence signals were recorded by ImageQuant LAS4000 hardware and analysed by ImageQuant LAS4000 software (both GE Healthcare, Chicago, IL, USA).

**Cell viability assay.** The cell viability was evaluated following *in vitro* treatment using Alexa Fluor^®^ 488 AnnexinV/Dead Cell Apoptosis Kit (Thermo Fisher Scientific, Waltham, MA, USA) following the manufacturer’s procedure. The samples were recorded on BD Accuri^TM^ C6 flow cytometer (Becton, Dickinson and Company, Franklin Lakes, NJ, USA) and analysed on FlowJo software (FlowJo version 10.2, LLC; Ashland, OR, USA).

**Cell cycle analysis.** The cells were washed in PBS, fixed/permeabilized with 70% ethanol, then treated with 20 μg/mL RNase (Sigma-Aldrich, Inc, St Louis, MO, USA) and 40 μg/mL PI (Sigma-Aldrich, Inc, St Louis, MO, USA). The samples were recorded on BD Accuri^TM^ C6 flow cytometer (Becton, Dickinson and Company, Franklin Lakes, NJ, USA) and analysed on FlowJo software (FlowJo version 10.2, LLC; Ashland, OR, USA).

**Uridine Uptake Assay.**The uridine uptake assay was carried out as described previously (Ladds et al, JBC) with modifications. Briefly, 200 000 ARN8 cells were seeded per well in 6-well plates in 2 mL of DMEM (Hyclone #SH30243) supplemented with 10% foetal bovine serum (FBS) v/v (Hyclone #SV30160) and 100 U mL^-1^ penicillin/streptomycin (Hyclone #SV30010). 24 hours post seeding, medium was removed and cells were treated with indicated compounds for 15 minutes in 0.7 mL of transport buffer (20 mM Tris HCl pH 7.4, 130 mM NaCl, 3 mM K2HPO_4_, 1 mM MgCl_2_, 5 mM glucose, 2 mM CaCl_2_). ^3^H uridine (Perkin Elmer #NET367001MC) was prepared in transport buffer to a final emission of 3.81 μCi mL^-1^ and added to cells for 60 seconds. After 60 seconds, the cells were washed with four quick changes of 1 mM unlabelled uridine prepared in ice-cold transport buffer. The samples were then harvested in 100 μL of 10% SDS. 900 μL of Optiphase Supermix (PerkinElmer #1200-439) was added to each sample and the counts per minute measured using a 1450 MicroBeta JET for liquid scintillation (PerkinElmer/Wallac).

**JeKo-1*^Luc+^* xenograft mouse model.** JeKo-1 cells were seeded at 5x10^5^ cells/mL in RPMI-1640 supplemented with 10% (v/v) HI-FBS, 1% (v/v) 200 mM L-Glutamine and 0.08‰ (w/v) polybrene. The cells were spinoculated for 90 minutes (1,000g at 32°C) with the RediFect Red-FLuc-GFP lentiviral particles (MOI = 1). Cells expressing high levels of GFP (JeKo-1*^Luc+^*) were sorted with the SH800Z cell sorter (Sony Biotechnology Inc., San Jose, CA, USA), using non-transduced JeKo-1 cells as a reference. JeKo-1*^Luc+^* cells were suspended in sterile saline at 2x10^6^ cells/mL. NOD-*scid*, NOD-*scid* β2m*^null^* and NOD-*scid* IL2rγ*^null^* mice (6–8 weeks old; Gades Institute, University of Bergen. NOD-*scid* IL2rγ*^null^* originally generous gift of Prof. Leonard D. Shultz, Jackson Laboratories, Bar Harbour, Maine, USA) were injected intravenously with 100 uL cell suspension (i.e. 2x10^5^ cells). The animals were assessed through weight registration, natural behaviour (grooming and nesting), the condition of the fur, lethargy, as well as the mouse grimace scale for evaluation of pain as described by Langford et al. Humane endpoint was reached when the mice lost > 10% body weight as compared to body weight at the initiation of the experiment, or if the general health status of the mice was reduced.

**Optical imaging.** Anaesthesia was induced by exposing the mice to 3% isoflurane and was maintained with 1 – 2 % isoflurane (Zoetis, Parsippany, NJ, USA) in 0.2 L/min oxygen. 150 mg/kg *D*-luciferin dissolved in sterile saline (B. Braun Melsungen AG, Melsungen, Germany) was administered intraperitoneally. The dorsal and ventral side of the mice were imaged 10 minutes post administration of *D*-luciferin. The bioluminescence signal was measured by IVIS Spectrum *In Vivo* Imaging System and the results were analysed by Living Image^®^ Software. To avoid dehydration of eyes under imaging, an eye ointment (simplex, Hydro Pharma) was applied.

**Combinatorial *in vivo* study.** 40 NOD-*scid* IL2Rγ*^null^* mice were inoculated intravenously with 2x10^5^ JeKo-1*^Luc+^* cells and divided into control, Bemcentinib (50 mg/kg, B.i.D.), Ibrutinib (25 mg/kg, Q.D.), (*R*)-HZ05 (75 mg/kg Q.D.) and combination of Bemcentinib and (*R*)-HZ05 (all *n* = 8). All groups were distributed based on bodyweight (one-way ANOVA; *p* = 0.9969) and total bioluminescence signal (one-way ANOVA; *p* = 0.9972). (*R*)-HZ05 was dissolved in a vehicle consisting of sterile water supplemented with 7.5% (v/v) DMSO, 45% (w/v) hydroxypropyl-beta-cyclodextrin and 25% (v/v) sterile saline to a final concentration of 7.5 mg/mL. The prepared compound was stored short-term at -20°C. The compound was administered by subcutaneous injections. Bemcentinib and Ibrutinib were dissolved in sterile water supplemented with 0.5% (w/v) hydroxypropyl methylcellulose and 0.1% (v/v) Tween^®^ 80 to a final concentration of 20 and 10 mg/mL, respectively. The compounds were administered by oral gavage. The control group was administered vehicle. All groups were treated 6 days x 4 weeks, starting on day 7 and were imaged on day 4 (pre-treatment), 11, 18, 25 and 32.

**Statistical analysis.** Statistical significance between different treatment groups was determined using a two-tailed Student *t*-test. Survival data were analysed by using a Log-rank (Mantle-Cox) test. To ensure no statistical significance between treatment groups, a one-way ANOVA was used. Relative half-maximal effective concentrations (EC50) were calculated in GraphPad PRISM^®^ 5.0 (GraphPad Software, La Jolla, CA, USA). Calculations of synergistic effects were performed in CompuSyn Software (CompuSyn Inc., New York, NY, USA). For all statistical analysis, *p <* 0.05 was considered significant.

**Supplemental references:**

Amin, H.M., McDonnell, T.J., Medeiros, L.J., Rassidakis, G.Z., Leventaki, V., O'Connor, S.L., Keating, M.J. & Lai, R. (2003) Characterization of 4 mantle cell lymphoma cell lines. *Arch Pathol Lab Med,* **127,** 424-431.
